# Anæsthetic Action of the Lycoperdon Proteus

**Published:** 1853-09

**Authors:** 


### MATERIA MEDICA AND THERAPEUTICS.

*Anæsthetic Action of the Lycoperdon Proteus.*—M. Flourens related some observations made by M. Gérard upon the anæsthetic action of the lycoperdon proteus. "I took," says the author, "the lycoperdons which I had gathered last autumn; I collected their substance, and then experimentalized with about twelve grammes (℥ss) of the capillitium, mixed with the spores. I put this substance, which burns like tinder, upon a match, which kept up the combustion, and plunged my head into the smoke for fifteen minutes. The sharpness of the smoke incommoded me at first, and caused a slight degree of irritation of the pharynx; then I had a coryza, which soon passed away; then a sensation of smarting in the eyes, which I was obliged to close; but the feeling of stupor was scarcely perceptible. During the inspiration of the smoke I withdrew from the apparatus, after the carbonization of the capillitium, and in a few instants I felt a sharp pain, weight of head, and a peri-cephalic constriction, without actual suffering. My eyes, which had become red, shut involuntarily, although there was no feeling of somnolence. After four hours, the head became free, but the malaise continued six hours. The day afterwards the conjunctiva was no longer red, but the irritation of the eyelids continued. I experienced none of the lethargy mentioned in the *Gazette des Hôpitaux*, where it is stated, that the animals submitted to its action fell into a condition resembling death.

I am sure that the properties of the lycoperdon bovista and L. excipulæ fermis are the same as those of the lycoperdon proteus."—*Med. Times and Gaz.*

